# Immunization routes in cattle impact the levels and neutralizing capacity of antibodies induced against *S. aureus* immune evasion proteins

**DOI:** 10.1186/s13567-015-0243-7

**Published:** 2015-09-28

**Authors:** Eveline Boerhout, Manouk Vrieling, Lindert Benedictus, Ineke Daemen, Lars Ravesloot, Victor Rutten, Piet Nuijten, Jos van Strijp, Ad Koets, Susanne Eisenberg

**Affiliations:** Ruminant Research and Development, MSD Animal Health, Wim de Körverstraat 35, 5830 AA Boxmeer, The Netherlands; Department of Infectious Diseases and Immunology, Faculty of Veterinary Medicine, Utrecht University, Yalelaan 1, 3584 CL Utrecht, The Netherlands; Department of Medical Microbiology, University Medical Center Utrecht, PO G04.614, Heidelberglaan 100, 3584 CX Utrecht, The Netherlands; Department of Farm Animal Health, Faculty of Veterinary Medicine, Utrecht University, Yalelaan 7, 3584 CL Utrecht, The Netherlands; Department of Veterinary Tropical Diseases, Faculty of Veterinary Science, University of Pretoria, Private Bag X04, Onderstepoort, 0110 South Africa; Department of Bacteriology and TSE, Central Veterinary Institute part of Wageningen UR, Edelhertweg 15, PO box 65, 8200 AB Lelystad, The Netherlands

## Abstract

Vaccines against *S. aureus* bovine mastitis are scarce and show limited protection only. All currently available vaccines are applied via the parenteral (usually intramuscular) route. It is unknown, however, whether this route is the most suitable to specifically increase intramammary immunity to combat *S. aureus* at the site of infection. Hence, in the present study, immunization via mucosal (intranasal; IN), intramuscular (triangle of the neck; IM), intramammary (IMM) and subcutaneous (suspensory ligament; SC) routes were analyzed for their effects on the quantity of the antibody responses in serum and milk as well as the neutralizing capacity of the antibodies within serum. The experimental vaccine comprised the recombinant *S. aureus* immune evasion proteins extracellular fibrinogen-binding protein (Efb) and the leukotoxin subunit LukM in an oil-in-water adjuvant combined with a hydrogel and alginate. The highest titer increases for both Efb and LukM specific IgG1 and IgG2 antibody levels in serum and milk were observed following SC/SC immunizations. Furthermore, the harmful effects of Efb and leukotoxin LukMF’ on host-defense were neutralized by serum antibodies in a route-dependent manner. SC/SC immunization resulted in a significant increase in the neutralizing capacity of serum antibodies towards Efb and LukMF’, shown by increased phagocytosis of *S. aureus* and increased viability of bovine leukocytes. Therefore, a SC immunization route should be considered when aiming to optimize humoral immunity against *S. aureus* mastitis in cattle.

**Electronic supplementary material:**

The online version of this article (doi:10.1186/s13567-015-0243-7) contains supplementary material, which is available to authorized users.

## Introduction

Infections with Staphylococci are common among humans and animals [1-3]. In cattle subclinical intramammary infections with *S. aureus* are common. Infections may lead to severe mastitis and/or chronic persistent infection with detrimental effects for the cows’ well-being, lifespan and milk production [4,5]. The current treatment of *S. aureus* infections with antibiotics often fails to completely clear the infection, due to specific cow or pathogen related risk factors [6]. Ineffective treatment may result in increased antibiotic resistance in *S. aureus*. Therefore, the availability of an effective vaccine would be of great value [7]. However, despite the numerous attempts to develop a highly efficacious vaccine, commercially available vaccines against *S. aureus* mastitis are scarce and evaluation under field conditions have shown to result in limited protection only [8]. All current vaccines are applied parenterally inducing a systemic immune reaction, which is reflected by an increase in specific antibodies in serum [9]. To reach the site of infection, antibodies induced by parenteral immunization need to be translocated to the milk and hence pass the blood-udder barrier, an effective, physiological separation between the systemic circulation and the udder tissue [10-13]. This does only occur once infection has been established, therefore the goal of preventing new intramammary infections has not been reached so far [14,15]. To develop an effective vaccine against *S. aureus* mastitis, it may be essential to increase intramammary, rather than systemic, humoral immunity. To date, little information is available regarding the impact of immunization routes on humoral immune responses in the bovine mammary gland [16,17].

From an immunological point of view, it is not clear whether the udder is part of the mucosal immune system or the skin immune system [18,19]. In addition, the environment of antigen uptake, processing and presentation may influence the magnitude of the antibody response as well as the neutralizing capacity of these antibodies. *S. aureus* expresses and secretes many immune evasion proteins [20]. Two of these proteins, extracellular fibrinogen-binding protein (Efb) and the leukotoxin subunit LukM, are suitable experimental antigens for the assessment of antibody quantity and their neutralizing capacity after immunization via different routes. Furthermore, both proteins are potential vaccine candidates since they are known to be involved in the pathogenesis of many *S. aureus* strains [21-24]. Efb is known to generate a capsule-like shield around the surface of *S. aureus* through a dual interaction with complement C3 and fibrinogen to mask surface-bound C3b and antibodies thereby escaping recognition by phagocytic cells like neutrophils [25]. LukM is the binding subunit of the bi-component leukotoxin LukMF’, competent of killing bovine peripheral blood leukocytes (PBLs) in a highly efficient manner [26,27]. Antibodies induced by immunization may prevent the interaction of Efb with C3, fibrinogen, or both, thereby preventing the formation of a capsule-like shield. In addition, neutralization of LukMF’ may be accomplished by antibodies blocking the interaction of LukM with its target receptor on the surface of neutrophils [28,29], or by antibodies blocking the required interaction between LukM and LukF’, thereby preventing pore formation [30]. Since it is thought that a delay in neutrophil lysis will allow these cells to phagocytose *S. aureus*, increased levels of neutralizing antibodies to both Efb and LukM are likely to improve vaccine efficiency [30,31].

The objective of this study was to analyze the impact of vaccine administration via different routes on the quantity of the antibody responses as well as the neutralizing capacity of these antibodies in dairy cattle using Efb and LukM as vaccine antigens.

## Materials and methods

### Animals

Sixteen clinically healthy mid-lactation dairy cows of the Holstein Frisian breed purchased in the Netherlands were housed at the Faculty of Veterinary Medicine (Utrecht, The Netherlands). After arrival, somatic cell counts (SCC) were determined at cow level by a commercial milk quality assurance laboratory (Qlip, Zutphen, The Netherlands). Only cows with a SCC <100 000 cells/mL were enrolled and were allowed an acclimatization period of two weeks to get used to the daily routine before sampling and immunization. Cows were fed a diet based on grass and corn silage, beet pulp and concentrate for the entire study period which was formulated to meet the dietary requirements for lactating dairy cows (Dutch feeding tables; [32]). Concentrate was administered via an automated feeding system and irregular concentrate uptake was monitored. Cows were milked twice a day and milk yield was recorded with an automated milk recording system. Following immunization, cows were daily monitored for signs of general and local reactions to the immunization by a veterinarian. The injection site was palpated to detect any swelling and painfulness. In cases where local reactions at the site of injection were observed a clinical examination of the cow was performed to identify general reactions. When immunization related changes in the udder tissue were detected, quarter level SCC was determined using the California mastitis test. When abnormalities in the milk were observed, bacterial culture was performed by plating 50 μL of milk onto sheep blood agar plates. After an overnight incubation at 37 °C bacterial growth was determined. Bacteria were presumptively identified by colony size, morphology, pigmentation and type of hemolysis.

The use of animals in this study was approved by the Ethical Committee for Animal Experiments of the Utrecht University (DEC2012.II.09.136) and conducted according to national regulations.

### Vaccine composition and recombinant proteins

The experimental vaccine consisted of an oil-in-water adjuvant combined with an alginate hydrogel (proprietary adjuvant, MSD-AH). As antigenic proteins, Efb and LukM, were used (50 μg/dose each). Cows were immunized with *S. carnosus* derived Efb and *E. coli* derived LukM. *S. carnosus* derived Efb was also used for ELISAs, while neutralization assays were performed with *E. coli* derived Efb, LukM and LukF’. For expression in *S. carnosus*, the gene encoding *efb* from the *S. aureus* Newbould305 strain (ATCC29740) was amplified by PCR and ligated into a pXR100 derived vector. *S. carnosus* culture supernatant was 0.2 μm filtered, analyzed on gel for Efb purity and concentration, and stored at −20 °C. For expression in *E. coli*, Efb, LukM and LukF’ proteins were generated as described previously [33,34]. Briefly, the *efb* gene of the *S. aureus* Newman strain and the *lukm* and *lukf* gene sequences of the *S. aureus* field isolate S1444 were amplified by PCR and ligated into the pRSETB vector (Invitrogen). The proteins were expressed with a six-residue N-terminal HIS-tag and purified by nickel-chelating chromatography (GE Healthcare) according to the manufacturer’s manual. Purified proteins were dialyzed against PBS and stored at −20 °C.

### Immunization

Cows were randomly assigned to four groups and immunized twice (1 mL/dose) with a 6 weeks interval. Immunizations were administered intranasal (IN/IN), intramuscular in the injection triangle of the neck (IM/IM), intramammary (one dose per each of the four milked-out quarters) followed by a subcutaneous booster close to the suspensory ligament (IMM/SC), and subcutaneous with both injections close to the suspensory ligament (SC/SC). The rationale behind the IMM/SC route was that cells primed in the mammary tissue would migrate to the local lymph node and re-enter the mammary gland following a booster immunization thereby enhancing the local immune response. For intranasal administration, aerosolized inoculums with a variable size were injected directly into the nostril using a nasal spray pump. For intramuscular and subcutaneous administrations 21G needles (BD Microlance™, Broek op Langedijk, The Netherlands) were used. For intramammary administrations a sterile plastic 5 mL-syringe and individual plastic infusion cannulas (Bovivet Animal Healthcare, Konannkunte, Banglore) were used.

### Sampling and sample preparation

Blood, milk, saliva and nasal secretion samples were collected at three time points: before immunization, 3 weeks after the priming immunization, and 2 weeks after the booster immunization. Blood was collected from the coccygeal vein using a sterile blood collection system (BD Vacutainer, Beckton Dickinson B.V., Breda, The Netherlands) and, after coagulation, centrifuged for 10 min at 1000 × *g* to collect serum. Milk samples, collected before the morning milking, were centrifuged for 10 min at 1000 × *g* to obtain skimmed milk. Saliva and nasal secretions were collected by inserting a tampon into the cow’s mouth or nostril. Tampons were removed after 1 min and transferred into a 20 mL syringe. To extract secretions, 4 mL of PBS (Lonza, Basel, Switzerland) were added and 1–2 mL of secretion was extracted from each tampon by compression within the syringe barrel. All samples were stored at −20 °C.

### ELISA

The presence of Efb and LukM specific IgG1, IgG2 and IgA antibodies in serum, milk, saliva and nasal secretions was determined by ELISA. Plates (NUNC MaxiSorp™, eBioscience, Affymetrix, Santa Clara, USA) were coated with 0.55 μg/mL Efb or 3 μg/mL LukM in 0.05 M sodium-bicarbonate buffer. Samples and positive control serum were tested in two-fold serial dilutions. An in-house negative control serum was taken along in eightfold. As secondary antibodies, horseradish-peroxidase-conjugated sheep α bovine IgG1, IgG2 and IgA (Bethyl Laboratories, Inc., Montgomery, USA) were used in 1:6000, 1:12 000 and 1:8000 dilutions for the Efb ELISAs, respectively. For the LukM ELISAs these antibodies were used in 1:4000, 1:8000 and 1:5000 dilutions, respectively. Tetramethylbenzidine was used as a substrate and reactions were stopped by adding 4 N sulphuric acid. Extinctions (450 nm) were measured on a Tecan SUNRISE™ (Tecan Group Ltd., Männedorf, Switserland) spectrophotometer using the XFluor4 Software Version V4.51-I4.

### Efb neutralization assay

The neutralizing capacity of specific Efb antibodies in serum was analyzed using a phagocytosis based assay described previously [25]. In short, 2.5 μg Efb was incubated with complement inactivated pre- or post-immunization serum or negative control serum for 10 min at room temperature (RT; 18–21 °C). Then, bovine lepirudin anti-coagulant plasma (5% final concentration; CSL Behring GmbH, Marburg, Germany) and 0.6 × 10^7^ CFU FITC-labeled *S. aureus* KV27 were added, followed by an incubation of 10 min at RT. Simultaneously, bovine PBLs of a blood donor where freshly isolated by adding 10 mL aquadest to 3 mL heparinized blood to lyse red blood cells. Then, 37 mL of RPMI medium (Gibco®, Paisley, Scotland) containing 0.05% human serum albumin was added followed by centrifugation for 4 min at 300 × *g*. Cells were washed twice and resuspended in medium. Leukocytes (5 × 10^5^ cells) were then added to the assay and phagocytosis was allowed for 15 min at 37 °C. The reaction was stopped by 1% phosphate-buffered formaldehyde fixation for 30 min at 4 °C and analyzed by flow cytometry on a FACSCalibur (Beckton Dickinson B.V., Breda, The Netherlands). To determine changes in phagocytosis as a result of antibody addition, cells were gated based on their forward and side scatter. The fluorescence of 10 000 gated neutrophils was measured for each sample. Phagocytosis was expressed as the percentage of neutrophils with a fluorescence above baseline (cells without fluorescent bacteria). Finally, phagocytosis ratios post- and pre-immunization were determined. The average ratio between post- and pre-immunization per cow was calculated from four independent experiments using blood from different donor cows.

### LukM neutralization assay

Complement inactivated pre- and post-immunization sera were analyzed for their ability to neutralize pore-formation and cell lysis induced by LukMF’. Sera were incubated with 33 nM LukMF’ for 30 min at RT and subsequently with 5 × 10^6^ bovine PBLs (isolated as described above) in the presence of 1.8 μg/mL 4’, 6-diamidino-2-phenulindole (DAPI). The final concentration of LukMF’ was 10 nM. DAPI fluorescence was measured in duplo for 30 min at 37 °C in a FLUOstar Omega microplate reader (BMG Labtech GMBH, Ortenberg, Germany). The time of lysis onset of the donor cells was defined as the time when DAPI-fluorescence reached three times the standard deviation of control samples that were incubated without LukMF’. The average time of lysis onset was calculated from three independent experiments.

### Calculations and statistics

Antibody titers were determined using CaSpEx Software AbendVertical version 0.11 V1 (MSD, Proprietary Software) and defined as the dilution of the sample that would give the same absorbance as the predefined cut-off. The cut-off was defined as 2* average negative control. Data analysis was performed in the SPSS statistical software package (version 20; IBM SPSS statistics 20.0; IBM Corp., Armonk, NY, USA). Data were checked for normality and Log2 transformed to achieve normality when necessary. Success of randomization was checked by comparing initial group antibody titers using an ANOVA with Bonferroni correction. Total titer increases and changes in phagocytic activity were calculated by subtracting pre-immunization values of post-booster values. Differences in total titer increase and percentage phagocytosis between groups were analyzed using the One-way ANOVA with Bonferroni correction. Differences in lysis onset pre- and post-immunization in the LukM neutralization assay were analyzed per group using a repeated measures analysis with the lysis onset as dependent variable. *P*-values below 0.05 were considered significant. For graphical presentation of the data GraphPad Prism software (Version 5; GraphPad Software Inc., La Jolla, CA, USA) was used.

## Results

### Clinical observations

The median lactation of enrolled cows was 2 (min 1; max 6). Cows produced on average 25.3 (SD ± 1.5) liters of milk per day throughout the study. According to the automated feeding system cows finished their daily concentrate ration during the entire study period. A small nodule at the site of injection developed in all IM and SC immunized cows during the first 3 days post-immunization. In one IMM immunized cow a hardened quarter with an increased SCC was observed one day following treatment. However, bacterial culture did not reveal the presence of any bacteria. IN immunized cows did not display any symptoms at all. No systemic reactions, changes in appetite or changes in milk production were observed throughout the study. All nodules at the application sites disappeared within 14 days following immunizations.

### Quantitative analyses of the antibody responses

**Figure 1:**
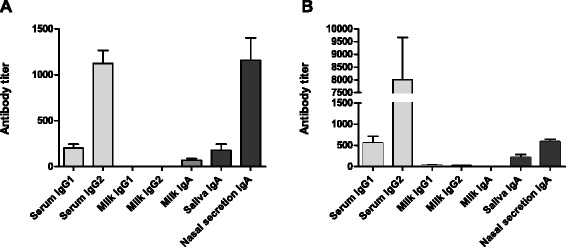
**Pre-immunization titers of isotype specific antibodies directed against Efb and LukM.** Initial Efb **(A)** and LukM **(B)** specific antibody titers in serum, milk, saliva and nasal secretion samples were measured by ELISA. Each bar represents mean ± SEM (*n* = 16).

Prior to immunization, in all cows Efb and LukM specific antibodies were detected in serum, milk, saliva and nasal secretions, with the exception of Efb specific IgG1 and IgG2, and LukM specific IgA antibodies in milk (Figure 1). The levels of these initial titers were not significantly different between groups (Additional file 1).

**Figure 2:**
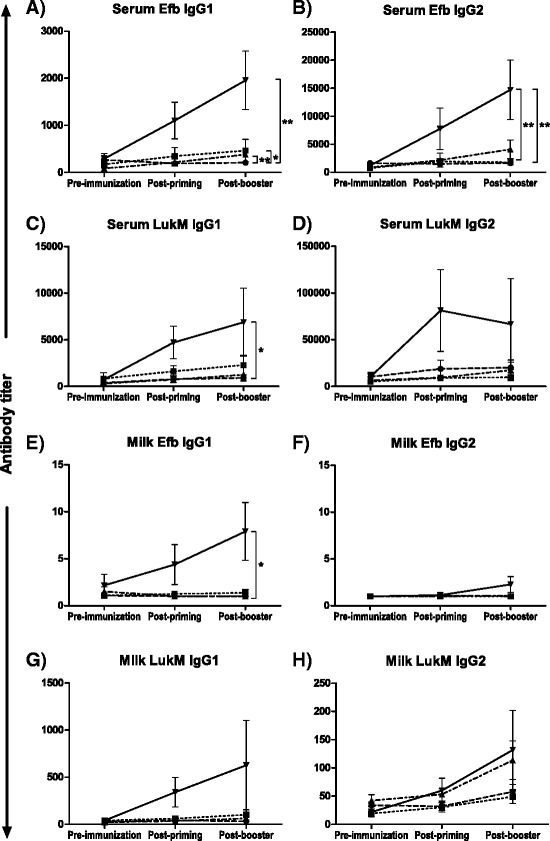
**IgG1 and IgG2 antibody titer increases in serum and milk.** Isotype specific antibody titers in serum **(A-D)** and milk **(E-H)** following IN/IN (●), IM/IM (■IMM/SC (▲) or SC/SC (▼) immunization with Efb **(A-B**, **E-F)** and LukM **(C**-**D**, **G**-**H)** were measured by ELISA. Results are expressed as the mean per group ± SEM. Differences in total titer increases following prime plus booster immunizations were analyzed using the One-way ANOVA. Letters indicate significant differences between IN/IN and IM/IM (*a*), IN/IN and IMM/SC (*b*), IN/IN and SC/SC (*c*), IM/IM and SC/SC (*d*), and IMM/SC and SC/SC (*e*) immunization routes.

Figure 2 shows the IgG1 and IgG2 antibody titer increases in serum and milk. Efb specific antibody titers in serum increased from 100 up to 2000 (IgG1) and from 1000 up to 15 000 (IgG2). LukM specific antibody titers in serum increased from 300 up to 7000 (IgG1) and from 6000 up to over 70 000 (IgG2). Milk titer increases were minimal for Efb specific IgG1 and IgG2 antibodies, whereas milk titer increases for LukM specific antibodies ranges from 50 to 600 (IgG1) and from 20 up to 130 (IgG2). No significant route specific increases in total antibody titers were observed over time. However, between routes statistically significant different titer increases were observed. Following priming and booster immunizations, the increase in Efb-specific IgG1 levels in serum was significantly higher in IM/IM, IMM/SC and SC/SC immunized animals than in IN/IN immunized cows (*p* = 0.037, *p* = 0.001 and *p* = 0.000, respectively; Figure 2A). The increase in Efb-specific IgG2 serum levels were higher in SC/SC immunized animals compared to IN/IN and IM/IM immunized cows (both *p <* 0.001; Figure 2B). Elevations in serum levels of LukM-specific IgG1 were also significantly higher following SC/SC immunizations compared to IN/IN immunizations (*p* = 0.041; Figure 2C). In contrast, route specific increases in LukM-specific IgG2 serum levels were not observed (Figure 2D). In milk, the increase in Efb-specific IgG1 was higher following SC/SC immunization than following IMM/SC immunization (*p* = 0.029; Figure 2E). These differences were not observed for Efb-specific IgG2 levels (Figure 2F). LukM-specific antibody titers in milk were only slightly affected by immunization via the different administration routes with the highest increases following SC/SC immunizations (Figure 2G–H). However, for all groups, increases in milk antibody levels compared to serum were only moderate and pre- and post-immunization titers were not statistically different.

In both milk and nasal secretions, Efb- and LukM-specific IgA levels were not affected by immunization. IgA levels in saliva were highly variable between cows and both increases and decreases were observed within groups in response to immunization (data not shown).

Serum and milk IgG1/IgG2 ratios did not change over the course of the experiment for both Efb and LukM, indicating that antibody increases following immunization with the experimental vaccine were similar for IgG1 and IgG2.

### Neutralizing capacity of the antibodies

**Figure 3:**
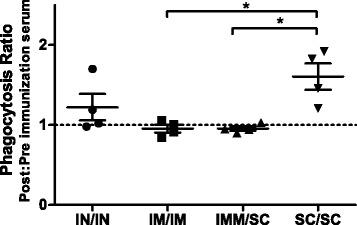
**Efb neutralization assay.** The presence of Efb neutralizing antibodies in serum of cows from different immunization groups were analyzed in an Efb neutralization assay. For each cow, the phagocytosis ratio between post- and pre-immunization serum was calculated from four independent neutralization experiments. Results are expressed as the mean ratio per group ± SEM. Differences in neutralization ratios between groups were calculated using the One-way ANOVA. *p < 0.05.

Antibodies within serum were tested for their ability to neutralize the inhibitory effects of Efb on phagocytosis. Figure 3 shows the differences in phagocytosis ratios of post- and pre-immunization serum. IM/IM and IMM/SC immunizations did not result in increased phagocytosis indicating that the induction of Efb neutralizing antibodies was limited. Only one cow immunized via IN/IN administration showed an increased percentage of phagocytosis post-immunization. In contrast, all SC/SC immunized animals showed an increase in neutralizing antibodies post-immunization. The increase in the Efb neutralizing capacity of serum antibodies was significantly higher following SC/SC immunization than after IM/IM and IMM/SC immunization (*p* = 0.015 for both groups).

**Figure 4:**
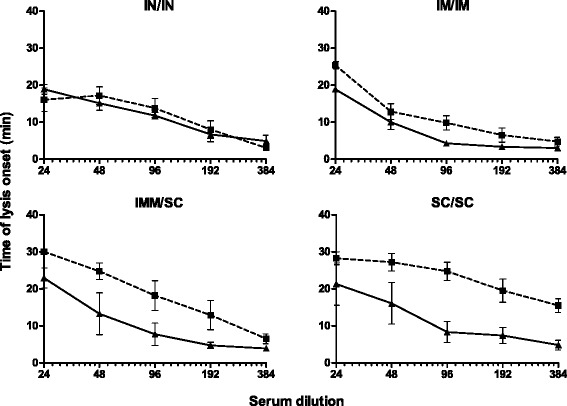
**LukMF’ neutralization assay.** The presence of LukMF’ neutralizing antibodies in serum of cows from different immunization groups were analyzed in a LukMF’ neutralization assay. For each group, the time of lysis onset between pre- (▲) and post- (■) immunization serum was calculated. Results are expressed as time of lysis onset ± SEM of three independent neutralization experiments. Differences in the time of lysis onset pre- and post-immunization within each immunization group were analyzed by repeated measures analysis. LukMF’ neutralization post-immunization was significantly increased in cows immunized via SC/SC (*p* = 0.009) and IM/IM (*p* = 0.012) administration when compared to pre- immunization.

Serum antibodies were also tested for their ability to neutralize the effects of LukM on PBL lysis. Figure 4 shows that LukM specific antibodies in serum of SC/SC immunized cows significantly blocked the pore forming ability of LukMF’ when compared to pre-immunization serum (*p* = 0.009). LukM specific antibodies in serum of IM/IM immunized cows also significantly decreased the pore formation by LukMF’ compared to pre-immunization serum (*p* = 0.012). However, the differences in neutralization pre- and post-immunization were limited compared to the increase observed in SC/SC immunized cows. A trend in LukMF’ neutralization was observed in serum of IMM/SC immunized cows (*p* = 0.058), whereas no differences in neutralization of LukMF’ were observed in serum of cows immunized via the IN/IN route.

## Discussion

The aim of this study was to assess whether the route of immunization impacts the quantity of the antibody response as well as the neutralizing capacity of these antibodies, with an emphasis on intramammary immunity to *S. aureus*.

Antibodies directed against Efb and LukM could be detected in all cows prior to immunization. The induction of antibodies against *S. aureus* immune evasion proteins following natural and experimentally induced acute and chronic infections has previously been described in human, mice, goats and cows [35-38]. The presence of these antibodies demonstrates that *S. aureus* expresses and secretes both proteins in vivo and that their presence can elicit an immune response. Since recurrent infections with *S. aureus* frequently occur, it is unlikely that the antibodies induced by natural infections are protective against *S. aureus*. The lack of protection (among other factors) may be due to insufficient antibody levels at the site of infection. Even though high initial IgG1 and IgG2 levels were detected in pre-immunization serum their levels in milk were remarkably lower. A lack of protection may also occur when naturally induced antibodies do, for the main part, not target relevant (i.e. neutralizing) epitopes. In this study, a rise in mainly serum, but also in milk antibody levels following SC/SC immunizations was observed. Furthermore, serum of SC/SC immunized cows showed increased neutralization capacity in in vitro neutralization assays. The increased antibody levels are likely to contribute to the increased neutralization observed post SC/SC immunization. These results are in line with previous studies where SC administration of recombinant Efb or LukM resulted in the induction of antibodies with a neutralizing effect on Efb and LukM in mice and rabbits [22,23]. The neutralizing capacity of antibodies in milk could not be measured since milk components interfere with the fluorescence based assays and insufficient amounts of milk were available for antibody isolation. As antibodies which are translocated over the blood-udder barrier retain their specificity [39,40], it is likely that the increased neutralizing capacity of Efb and LukM specific antibodies is also present in milk. The influence of immunizations on IgA levels in saliva and nasal secretions were highly variable. This might be due to the sample collection technique used since the volumes of saliva and nasal secretions collected in the tampons were not controlled during sampling. This may have influenced the final antibody concentration of the analyzed fluids. Therefore, a more standardized method is required in order to analyze the IgA response following immunization. Whether neutralizing antibodies are beneficial to the clearance of *S. aureus* from the udder at all has to be established in future research.

The immunization route and the applied adjuvant determine the immunological environment antigens are taken up and processed in [41]. Different responses may be elicited when antigens are administrated in different tissues [42,43]. Cytokines in the local environment influence the process of T cell stimulation by dendritic cells and eventually determine the antibody isotype produced by B-cells [40]. In this study, no shift in IgG1/IgG2 ratios was observed following immunization, indicating a similar production of both antibody isotypes regardless of the immunization route. These findings correspond with earlier studies where cows were immunized with an *S. aureus* capsular polysaccharide type 5 conjugate in combination with a mineral oil adjuvant or with a polysaccharide from *Streptococcus agalactiae* conjugated to ovalbumin in Freund’s incomplete adjuvant [44-46]. However, two other studies found a more pronounced IgG2 response after immunization with an *S. aureus* CP5-ovalbumin conjugate in Freund’s incomplete adjuvant or with a killed *S. aureus* cell-toxoid vaccine using dextran sulphate as an adjuvant [47,48]. Since alveolar macrophages in the udder have been shown to lack IgG2 receptors [49] it has been speculated that, in order to enhance local immunity, it is preferable to increase the IgG1 titer in milk. IgG1 is selectively transported to the mammary gland by the FcRn receptor [45]. During the development of infection IgG2 leaks into the milk together with other serum components [50]. Therefore, IgG2 is thought to be more important in the second line of defense. Notwithstanding, in the study presented here immunization with antigens in a modified oil-in-water adjuvant via different administration routes did resulted in different levels and neutralizing capacity of antibodies. Of all administration routes applied, subcutaneous tissue exhibited the best environment to evoke a response against both Efb and LukM. Since all SC injections were administered close to the suspensory ligament it cannot be distinguished whether the subcutaneous route as such was responsible for this beneficial effect or whether it was the regional administration that influenced the humoral response. However, previous studies did not observe differences in serum and milk antibody titer increases in cows immunized via SC administration in the neck versus SC administration in the area of the supramammary lymph node indicating that the administration route might be more important than the location of administration [51,52].

Intranasal, mucosal immunization resulted in a marginal response only, indicating that this route is less suitable for generating humoral protection against *S. aureus* immune evasion proteins in the presence of the chosen adjuvant. Although the classical IM route is widely used and described to stimulate the humoral immune response in a similar way as the SC route, in this study it only led to an intermediate increase in antibody titers as did the combined IMM/SC route [53,54].

In conclusion, this study showed that immunization routes impact the antibody response induced against the *S. aureus* immune evasion proteins Efb and LukM. A subcutaneous immunization in the suspensory ligament region resulted in higher antibody levels with increased neutralization capacities when compared to the other immunization routes.

## Additional file

Additional file 1:
**Initial antibody titers per group.** Prior to immunization, Efb and LukM specific antibody titers in serum, milk, saliva and nasal secretion samples were measured by ELISA. Results are presented as the mean antibody titer per group (Log2) ± SEM. Success of randomization was checked by comparing initial group antibody titers using an ANOVA with Bonferroni correction. The levels of initial antibody titers were not significantly different between groups.

## References

[1] Boucher H, Miller LG, Razonable RR (2010). Serious infections caused by methicillin-resistant Staphylococcus aureus. Clin Infect Dis.

[2] Foster AP (2012). Staphylococcal skin disease in livestock. Vet Dermatol.

[3] Holmes MA, Zadoks RN (2011). Methicillin resistant S. aureus in human and bovine mastitis. J Mammary Gland Biol Neoplasia.

[4] Keefe G (2012). Update on control of Staphylococcus aureus and Streptococcus agalactiae for management of mastitis. Vet Clin North Am Food Anim Pract.

[5] Peton V, Le Loir Y (2014). Staphylococcus aureus in veterinary medicine. Infect Genet Evol.

[6] Sears PM, McCarthy KK (2003). Management and treatment of staphylococcal mastitis. Vet Clin North Am Food Anim Pract.

[7] Smith GW, Lyman RL, Anderson KL (2006). Efficacy of vaccination and antimicrobial treatment to eliminate chronic intramammary Staphylococcus aureus infections in dairy cattle. J Am Vet Med Assoc.

[8] Schukken YH, Bronzo V, Locatelli C, Pollera C, Rota N, Casula A, Testa F, Scaccabarozzi L, March R, Zalduendo D, Guix R, Moroni P (2014). Efficacy of vaccination on Staphylococcus aureus and coagulase-negative staphylococci intramammary infection dynamics in 2 dairy herds. J Dairy Sci.

[9] Pereira UP, Oliveira DG, Mesquita LR, Costa GM, Pereira LJ (2011). Efficacy of Staphylococcus aureus vaccines for bovine mastitis: a systematic review. Vet Microbiol.

[10] Hurley WL, Theil PK (2011). Perspectives on immunoglobulins in colostrum and milk. Nutrients.

[11] Mayer B, Doleschall MF, Bender BF, Bartyik J, Bosze Z, Frenyó LV, Kacskovics I (2005). Expression of the neonatal Fc receptor (FcRn) in the bovine mammary gland. J Dairy Res.

[12] Nguyen DA, Neville MC (1998). Tight junction regulation in the mammary gland. J Mammary Gland Biol Neoplasia.

[13] Stelwagen K, Carpenter E, Haigh BF, Hodgkinson AF, Wheeler TT (2009). Immune components of bovine colostrum and milk. J Anim Sci.

[14] Middleton JR, Luby CD, Adams DS (2009). Efficacy of vaccination against staphylococcal mastitis: a review and new data. Vet Microbiol.

[15] Tiwari JG, Babra C, Tiwari HK, Williams V, de Wet S, Gibson J, Paxman A, Morgan E, Costantino P, Sunagar R, Isloor S, Mukkur T (2013). Trends in therapeutic and prevention strategies for management of bovine mastitis: an overview. J Vaccines Vaccin.

[16] Finch JM, Hill AW, Field TR, Leigh JA (1994). Local vaccination with killed Streptococcus uberis protects the bovine mammary gland against experimental intramammary challenge with the homologous strain. Infect Immun.

[17] Wilson DJ, Gonzalez RN (2003). Vaccination strategies for reducing clinical severity of coliform mastitis. Vet Clin North Am Food Anim Pract.

[18] Dosogne H, Vangroenweghe F, Burvenich C (2002). Potential mechanism of action of J5 vaccine in protection against severe bovine coliform mastitis. Vet Res.

[19] Vorbach C, Capecchi MR, Penninger JM (2006). Evolution of the mammary gland from the innate immune system?. Bioessays.

[20] Foster TJ, Geoghegan JA, Ganesh VK, Höök M (2014). Adhesion, invasion and evasion: the many functions of the surface proteins of Staphylococcus aureus. Nat Rev Microbiol.

[21] Garcia BL, Ramyar KX, Ricklin D, Lambris JD, Geisbrecht BV (2012). Advances in understanding the structure, function, and mechanism of the SCIN and Efb families of Staphylococcal immune evasion proteins. Adv Exp Med Biol.

[22] Padmaja RJ, Halami PM (2014). Immunogenicity of Staphylococcus aureus LukM/F’-PV recombinant subunits: validation of diagnostic potential and evaluation of protective efficacy in vitro. Vet Microbiol.

[23] Shannon O, Uekotter AF, Flock JI (2006). The neutralizing effects of hyperimmune antibodies against extracellular fibrinogen-binding protein, Efb, from *Staphylococcus aureus*. Scand J Immunol.

[24] Zecconi A, Binda E, Borromeo VF, Piccinini R (2005). Relationship between some Staphylococcus aureus pathogenic factors and growth rates and somatic cell counts. J Dairy Res.

[25] Ko YP, Kuipers A, Freitag CM, Jongerius I, Medina E, van Rooijen WJ, Spaan AN, van Kessel KP, Höök M, Rooijakkers SH (2013). Phagocytosis escape by a Staphylococcus aureus protein that connects complement and coagulation proteins at the bacterial surface. PLoS Pathog.

[26] Fromageau A, Gilbert FB, Prévost G, Rainard P (2010). Binding of the Staphylococcus aureus leucotoxin LukM to its leucocyte targets. Microb Pathog.

[27] Vrieling M, Koymans KJ, Heesterbeek DA, Aerts PC, Rutten VP, de Haas CJ, van Kessel KP, Koets AP, Nijland R, van Strijp JA (2015). Bovine Staphylococcus aureus secretes the leukocidin LukMF’ to kill migrating neutrophils through CCR1. MBio.

[28] Barrio MB, Rainard PF, Prevost G (2006). LukM/LukF’-PV is the most active Staphylococcus aureus leukotoxin on bovine neutrophils. Microbes Infect.

[29] Spaan AN, Henry TF, van Rooijen WJ, Perret M, Badiou C, Aerts PC, Kemmink J, de Haas CJ, van Kessel KP, Vandenesch F, Lina G, van Strijp JA (2013). The staphylococcal toxin Panton-Valentine Leukocidin targets human C5a receptors. Cell Host Microbe.

[30] Yoong P, Torres VJ (2013). The effects of *Staphylococcus aureus* leukotoxins on the host: cell lysis and beyond. Curr Opin Microbiol.

[31] Spaan AN, Surewaard BG, Nijland RF, van Strijp JA (2013). Neutrophils versus *Staphylococcus aureus*: a biological tug of war. Annu Rev Microbiol.

[32] Diervoeder P (2012). CVB Tabellenboek Veevoeding.

[33] Ko YP, Liang XF, Smith CW, Degen JL, Hook M (2011). Binding of Efb from *Staphylococcus aureus* to fibrinogen blocks neutrophil adherence. J Biol Chem.

[34] Jongerius I, Kohl JF, Pandey MK, Ruyken M, van Kessel KP, van Strijp JA, Rooijakkers SH (2007). Staphylococcal complement evasion by various convertase-blocking molecules. J Exp Med.

[35] Colque-Navarro P, Palma M, Söderquist B, Flock JI, Möllby R (2000). Antibody responses in patients with staphylococcal septicemia against two *Staphylococcus aureus* fibrinogen binding proteins: clumping factor and an extracellular fibrinogen binding protein. Clin Diagn Lab Immunol.

[36] Jongerius I, Kockritz-Blickwede MF, Horsburgh MJ, Ruyken MF, Nizet VF, Rooijakkers SH (2012). *Staphylococcus aureus* virulence is enhanced by secreted factors that block innate immune defenses. J Innate Immun.

[37] Rainard P (2014). Staphylococcus aureus leucotoxin LukM/F’ is secreted and stimulates neutralising antibody response in the course of intramammary infection. Vet Res.

[38] Verkaik NJ, Lebon AF, de Vogel CP, Hooijkaas H, Verbrugh HA, Jaddoe VW, Hofman A, Moll HA, van Belkum A, van Wamel WJ (2010). Induction of antibodies by *Staphylococcus aureus* nasal colonization in young children. Clin Microbiol Infect.

[39] Acres SD, Isaacson RE, Babiuk LA, Kapitany RA (1979). Immunization of calves against enterotoxigenic colibacillosis by vaccinating dams with purified K99 antigen and whole cell bacterins. Infect Immun.

[40] Dudek K, Bednarek D, Ayling RD, Szacawa E (2014). Stimulation and analysis of the immune response in calves from vaccinated pregnant cows. Res Vet Sci.

[41] Coffman RL, Sher A, Seder RA (2010). Vaccine adjuvants: putting innate immunity to work. Immunity.

[42] Holmgren J, Czerkinsky C (2005). Mucosal immunity and vaccines. Nat Med.

[43] Pulendran B, Banchereau J, Maraskovsky E, Maliszewski C (2001). Modulating the immune response with dendritic cells and their growth factors. Trends Immunol.

[44] Tollersrud T, Zernichow LF, Andersen SRFAU, Kenny KF, Lund A (2001). Staphylococcus aureus capsular polysaccharide type 5 conjugate and whole cell vaccines stimulate antibody responses in cattle. Vaccine.

[45] Takimori S, Shimaoka H, Furukawa J, Yamashita T, Amano M, Fujitani N, Takegawa Y, Hammarström L, Kacskovics I, Shinohara Y, Nishimura S (2011). Alteration of the N-glycome of bovine milk glycoproteins during early lactation. FEBS J.

[46] Rainard P (1992). Isotype antibody response in cows to Streptococcus agalactiae group B polysaccharide-ovalbumin conjugate. J Clin Microbiol.

[47] Gilbert FB, Poutrel BF, Sutra L (1994). Immunogenicity in cows of Staphylococcus aureus type 5 capsular polysaccharide-ovalbumin conjugate. Vaccine.

[48] Watson DL (1992). Vaccination against experimental staphylococcal mastitis in dairy heifers. Res Vet Sci.

[49] Howard CJ, Taylor G, Brownlie J (1980). Surface receptors for immunoglobulin on bovine polymorphonuclear neutrophils and macrophages. Res Vet Sci.

[50] Mackenzie DD, Lascelles AK (1968). The transfer of [131-I]-labelled immunoglobulins and serum albumin from blood into milk of lactating ewes. Aust J Exp Biol Med Sci.

[51] Nickerson SC, Owens WE, Boddie RL (1993). Effect of a *Staphylococcus aureus* bacterin on serum antibody, new infection, and mammary histology in nonlactating dairy cows. J Dairy Sci.

[52] Tomita GM, Nickerson SC, Owens WE, Wren B (1998). Influence of route of vaccine administration against experimental intramammary infection caused by Escherichia coli. J Dairy Sci.

[53] Bradley AJ, Breen JE, Payne B, White V, Green MJ (2015). An investigation of the efficacy of a polyvalent mastitis vaccine using different vaccination regimes under field conditions in the United Kingdom. J Dairy Sci.

[54] Gurjar AA, Klaessig S, Salmon SA, Yancey RJ, Schukken YH (2013). Evaluation of an alternative dosing regimen of a J-5 mastitis vaccine against intramammary *Escherichia coli* challenge in nonlactating late-gestation dairy cows. J Dairy Sci.

