# Supplementary material for: Immunization routes in cattle impact the levels and neutralizing capacity of antibodies induced against S. aureus immune evasion proteins
Source: Vet Res. 2015 Sep 28;46:115. doi: 10.1186/s13567-015-0243-7 (PMC4584483; doi:10.1186/s13567-015-0243-7)
Supplement: Additional file 1: — Initial antibody titers per group. Prior to immunization, Efb and LukM specific antibody titers in serum, milk, saliva and nasal secretion samples were measured by ELISA. Results are presented as the mean antibody titer per group (Log2) ± SEM. Success of randomization was checked by comparing initial group antibody titers using an ANOVA with Bonferroni correction. The levels of initial antibody titers were not significantly different between groups. [file 13567_2015_243_MOESM1_ESM.docx]

|  |  |  | **Administration** **route** | | | |  |
| --- | --- | --- | --- | --- | --- | --- | --- |
| **Antigen** | **Specimen** | **Isotype** | **IN/IN** | **IM/IM** | **IMM/SC** | **SC/SC** | ***p*-value** |
| Efb | Serum | IgG1 | 7.78 *± 1.09* | 6.95 *± 1.32* | 6.08 *± 1.14* | 7.48 *± 2.05* | 0.406 |
|  |  | IgG2 | 10.65 *± 0.29* | 9.40 *± 0.91* | 9.45 *± 1.31* | 10.05 *± 0.90* | 0.240 |
|  |  |  |  |  |  |  |  |
|  | Milk | IgG1 | 0.10 *± 0.20* | 0.10 *± 0.20* | 0.40 *± 0.80* | 0.63 *± 1.25* | 0.303 |
|  |  | IgG2 | 0.00 *± 0.00* | 0.00 *± 0.00* | 0.00 *± 0.00* | 0.00 *± 0.00* | - |
|  |  | IgA | 6.73 *± 1.48* | 5.25 *± 1.20* | 4.83 *± 1.02* | 5.30 *± 1.04* | 0.182 |
|  |  |  |  |  |  |  |  |
|  | Saliva | IgA | 6.95 *± 1.51* | 5.58 *± 1.84* | 6.53 *± 1.34* | 6.70 *± 2.26* | 0.715 |
|  |  |  |  |  |  |  |  |
|  | Nasal secretion | IgA | 8.45 *± 0.40* | 9.53 *± 0.25* | 9.18 *± 0.92* | 9.18 *± 0.40* | 0.051 |
| LukM | Serum | IgG1 | 8.25 *± 1.09* | 8.53 *± 2.03* | 7.85 *± 1.30* | 9.13 *± 1.37* | 0.678 |
|  |  | IgG2 | 13.00 *± 1.00* | 12.13 *± 0.88* | 12.35 *± 1.18* | 12.80 *± 1.49* | 0.703 |
|  |  |  |  |  |  |  |  |
|  | Milk | IgG1 | 4.50 *± 1.22* | 4.78 *± 1.48* | 4.15 *± 1.05* | 5.20 *± 0.94* | 0.622 |
|  |  | IgG2 | 5.00 *± 0.59* | 3.83 *± 1.56* | 5.28 *± 0.65* | 4.35 *± 0.62* | 0.904 |
|  |  | IgA | 1.15 *± 0.17* | 1.45 *± 0.77* | 1.78 *± 0.94* | 1.73 *± 0.84* | 0.627 |
|  |  |  |  |  |  |  |  |
|  | Saliva | IgA | 7.38 *± 1.79* | 6.6 *± 1.91* | 7.65 *± 1.59* | 5.48 *± 1.08* | 0.280 |
|  |  |  |  |  |  |  |  |
|  | Nasal secretion | IgA | 8.28 *± 1.77* | 9.68 *± 1.24* | 9.38 *± 1.03* | 11.08 *± 0.62* | 0.097 |
